# Atypical cerebellar activity and connectivity during affective touch in adults with skin-picking disorder

**DOI:** 10.1007/s11682-023-00824-z

**Published:** 2023-11-17

**Authors:** Albert Wabnegger, Anne Schienle

**Affiliations:** Clinical Psychology, University of Graz, BioTechMed, Universitätsplatz 2/III, Graz, A-8010 Austria

**Keywords:** Skin-picking disorder, Affective touch, Lobule VIII activity/ connectivity, Motor control, Reward processing

## Abstract

Excessive touching and picking of one’s skin are core symptoms of skin-picking disorder (SPD). Previous research has shown that patients with SPD display difficulties in motor control and show altered reward responsivity. Considering the limited knowledge about neuronal mechanisms in SPD, particularly concerning the cerebellum, the analysis focused on this brain region due to its involvement in sensorimotor and affective functions. The participants of the present study received affective (caress-like), which is typically perceived as pleasant and can be passively enjoyed. A total of 132 female participants (70 patients with SPD, 62 healthy controls) received affective and nonaffective touch to their forearms (slow vs. fast brushing) during functional magnetic resonance imaging. This tactile stimulation was rated according to pleasure, arousal, and the urge to pick one’s skin. Being touched was perceived as more negative and arousing by the SPD group, and elicited a greater urge to perform skin-picking. During affective touch, those participants with SPD were characterized by reduced activity in lobule VIII, reduced functional connectivity of lobule VIII with the hippocampus, and increased connectivity with the superior parietal lobule. Since VIII is involved in the inhibition of movement, the present findings point to deficient motor control in SPD in the context of affective-sensory processing.

**Supplementary Information:**

The online version contains supplementary material available at 10.1007/s11682-023-00824-z.

## Introduction

The neuropathological mechanisms of skin-picking (excoriation) disorder (SPD) are still poorly understood. Repeated and excessive touching, scratching, picking, and digging into the own skin are core symptoms of SPD (DSM-5, APA, 2013). Picking usually involves fingertips/fingernails and aims at the removal of skin irregularities (e.g., pimples, patches of dry skin, or scabbed-over cuts and scratches). The majority of patients with SPD experience the picking as pleasant (e.g., calming, rewarding; Gallinat et al., 2021; Schienle et al., 2018b), although the consequences of this ‘compulsive’ behavior are negative (tissue damage, distress, and functional impairment; APA, 2013).

Thus far, functional neuroimaging studies on SPD have mainly identified two behavioral dimensions and their neural correlates that are associated with pathological skin-picking: affective processing (reward/punishment) and motor control (for a review see Torales et al., 2020). For example, when presented with disorder-specific visual stimuli (images with skin irregularities) individuals with SPD reported increased negative affect and displayed increased activation in the amygdala and insula (Schienle et al., 2018a). Other fMRI studies revealed general dysfunctions in the processing of reward and punishment related to SPD (e.g., Wabnegger et al., 2018; Grant et al., 2022). The participants of these studies were exposed to negative affective pictures, or anticipated monetary reward/ punishment. Differences in activity between patients with SPD and controls were observed in regions concerned with emotional processing (insula, amygdala), and motor control (inferior frontal gyrus, anterior cingulate cortex). By analyzing resting-state functional connectivity data, Huggins et al. (2020) found the supplementary motor area (SMA) to be associated with skin-picking symptom severity. The SMA is involved in the preparation of motor actions.

A brain region that is related to both affective and motor functions is the cerebellum. This brain region has been traditionally viewed as primarily involved in sensorimotor functions. However, neuroimaging studies have provided evidence that the cerebellum also modulates emotional processes (recognition, experience, and regulation of emotional states and social behaviors). A meta-analysis by Stoodley and Schmahmann (2009) identified lobules VI, Crus I, and the medial part of lobule VII of the cerebellum as central subregions to mediate affective functions. Sensorimotor tasks activate the anterior lobe of the cerebellum (lobules I-V).

Interestingly, only two neuroimaging studies have reported atypical cerebellar activity for patients with SPD (Wabnegger et al., 2019; Grant et al., 2022). In one study, Wabnegger et al. (2019) asked the participants to caress (vs. scratch) a selected skin area on their forearms (‘self-touch’). During caressing, patients displayed decreased activation in Crus I, as well as decreased coupling of this area with the ventrolateral prefrontal cortex, relative to healthy controls. This finding points to SPD-related dysfunctional reward processing in the context of tactile stimulation.

The present study followed up on this finding and investigated cerebellar activity in individuals with SPD during the administration of another type of tactile stimulation. Instead of caressing themselves, this time the participants were touched by another person and received affective touch. Affective touch refers to gentle and slow (1–10 cm/s) stroking of the skin that is generally perceived as caress-like and pleasant (Cruciani et al., 2021). A meta-analysis (Morrison, 2016) demonstrated that affective touch is associated with activation in the primary and secondary somatosensory cortex, prefrontal cortex regions, and the insula. Activation in the cerebellum was not reported.

Due to the limited database concerning the involvement of the cerebellum in the symptomatology of SPD, the present study with 132 participants (70 patients with SPD and 62 healthy controls) followed a mainly exploratory approach. It was investigated whether SPD patients would show altered cerebellar activity and connectivity during a rewarding tactile stimulation (interpersonal affective touch). The analysis focused on those cerebellar regions that are implicated in emotional (e.g., lobule V), and motor processing (e.g., lobule VIII; see Stoodley & Schmahman, 2009).

## Materials and methods

### Participants

Seventy female patients with a primary DSM-5 diagnosis of skin-picking disorder (SPD group; age range: 19–59 years) and 62 healthy females (Control group; CG; age range: 18–60 years) participated in this study. The SPD diagnosis had been made by a board-certified clinical psychologist based on a clinical interview (Yale–Brown Obsessive Compulsive Scale Modified for Neurotic Excoriation).

Exclusion criteria for the SPD group were diagnoses of major depression with severe symptoms, substance abuse/ dependence, borderline personality disorder, psychosis, and dermatological conditions (e.g., scabies, and psoriasis). Additional comorbidities (in 47% of the patients) did not lead to exclusion: anxiety disorders (generalized anxiety disorder, panic disorder, specific phobia; 36%); depression (mild to moderate symptoms; 4%); obsessive-compulsive disorder (3%), and eating disorders (6%).

Exclusion criteria for the control group were reported diagnoses of mental disorders and dermatological conditions.

A statistical power analysis indicated that for an effect size of f = 0.16, with a power of 0.95 and an alpha level of 0.05 for a mixed-model analysis of variance (two between-subjects factors, two within-subjects factors; correlation between repeated measures = 0.5) 130 participants would be needed (G*Power 3.1; Faul et al., 2009).

The sample was restricted to females because of sex differences concerning the prevalence of SPD and affective touch processing (APA, 2013; Grant & Chamberlain, 2020; Jönsson et al., 2017). The participants were recruited via social media and the outpatient clinic of the department.

### Questionnaires

Participants completed the Skin-Picking Scale revised (SPS-R; Cronbach’s alpha in the present sample α = 0.96) and the Milwaukee Inventory for the Dimensions of Adult Skin Picking (MIDAS; α = 0.84). The SPS-R is a questionnaire to assess the severity of skin-picking symptoms. The MIDAS measures focused skin-picking (ritualized skin manipulation) and automatic skin-picking (skin manipulation outside of conscious awareness).

### Procedure

The participants were invited to a study focusing on touch processing. For the fMRI experiment, a well-validated study design for affective touch was implemented, whereby an experimenter administered slow and fast brush strokes to the forearm of participants (Cruciani et al., 2021). The tactile stimulation was administered by a trained female research assistant, who used a hand-held soft boar bristle brush. Slow and fast brushing was guided by a metronome (via headphones). Slow touch had a velocity of 3 cm/s and an approximate indentation force of 0.3 N on the forearm (stroking in proximal to distal direction, 8 cm region, marked by two tape stripes), whereas fast brushing had a velocity of 30 cm/s. The two brushing conditions (slow/fast) lasted for 6 s. Each condition was repeated 12 times interspersed with rest blocks (no brushing for 12 s). The sequence of the brushing conditions was randomized.

After each condition, the participants rated the pleasantness of the touch (valence), their arousal, and their urge to perform skin-picking on 9-point visual analog scales (1 = not pleasant/aroused/no urge; 9 = very pleasant/aroused/ strong urge). The order of the ratings was constant across the experiment.

The participants had their eyes closed during the brushing. A first signal tone (presented for 2 s) after each condition indicated to open the eyes and respond to the visually presented rating scales (12 s). The verbal ratings were recorded via a scanner-suitable device. A second signal tone (2 s) indicated closing the eyes for the subsequent brushing condition.

The study complied with all relevant ethical guidelines and regulations involving human participants and was approved by the ethics committee of the University of Graz, Austria (GZ 39/29/26 ex 2018/19). All participants provided informed consent before participating. This study was preregistered on the German Clinical Trials Register (DRKS00022123, 06/08/2020). Findings on cerebral activation during affective touch in the studied sample have been reported elsewhere, Schienle et al., 2023). Relative to healthy controls, patients with SPD showed altered activity in frontal and parietal regions of interest (supramarginal/ angular gyrus, middle/ inferior frontal gyrus) involved in attentional control. The whole-brain analysis revealed no group differences.

### Recording and analysis of fMRI data

The MRI session was conducted with a 3 T scanner (Vida, Siemens, Erlangen, Germany) with a 64-channel head coil. Functional runs were acquired using a T2*-weighted multiband EPI protocol (number of slices: 58, interleaved, flip angle = 82°, slice thickness: 2.5 mm; slice spacing: 2.5 mm; TE = 0.03 s; TR = 1800 ms; multi-band accel. factor = 2; acquisition matrix: 88; in-plane resolution = 2.5 × 2.5 × 2.5 mm). Structural images were obtained using a T1-weighted MPRAGE sequence (voxel size: 1 × 1 × 1 mm; acquisition matrix: 224, slice thickness: 1 mm, TE = 0.00236, TR = 1600 ms; flip angle = 9°). All analyses were conducted with SPM12 (version: 7487; Wellcome Department of Cognitive Neurology, London), the SUIT toolbox (version 3.3), and the generalized PsychoPhysiological Interactions toolbox (gPPI; McLaren et al., 2012).

Four participants from the SPD group had to be excluded from the analysis due to motion artifacts (n = 2) and scanner-related artifacts (n = 2), leaving 128 data sets.

To investigate isolated cerebellar activity and cerebellum-cerebrum connectivity we used two separate preprocessing streams similar to previous work (e.g., Mehnert et al., 2017). First, preprocessing of whole-brain functional data comprised motion correction by realignment and unwarping (registering to the first image) followed by slice timing (reference slice = middle). Realigned and slice-time corrected time series were then forwarded into the first-level analysis in the subject’s native space. The regressors ‘rating_scale’ ‘slow’ and ‘fast’ were entered and convoluted with the canonical hemodynamic response function together with the six motion parameters and the contrast ‘slow-fast’ was built. Moreover, we performed calculations to determine the overall displacement for each participant, thereby generating an individual metric reflecting the degree of motion exhibited during the scanning process. This metric was subsequently compared between both groups and correlated with the rating data. Additionally, an AR(1) process was applied to account for biorhythms and unmodeled neural activity, and the high-pass filter was set to 175.

For investigating cerebellar activity, the cerebellum and brainstem were first isolated from the whole brain T1-weighted image (origin set to the AC-PC line) for each individual with the help of the SUIT toolbox. As other non-cerebellar parts (e.g., the transverse sinus) were misclassified as parts of the cerebellum, an additional manual correction of the individual isolation map was necessary for most individuals. The segmented anatomical cerebellum in native space was normalized to the SUIT template using a nonlinear deformation. To obtain isolated cerebellar activity, the whole brain contrast images were then resliced using the deformation maps generated in the previous step. This masks out activity outside the cerebellum or brainstem. These normalized contrast images (voxel size 2 mm isotropic) were finally smoothed with an isotropic 4 mm FWHM (field width at half maximum) and forwarded to a second-level general linear model (GLM).

Cerebellar activity between both groups for the contrast ‘slow – fast’ was compared using a two-sample t-test. Furthermore, to compare the slopes (i.e., betas) of the relationship between rating and brain data between both groups we calculated difference scores for the rating data (e.g., slow_arousal – fast_arousal), which were then used as a predictor in a two-sample t-test (interaction with factor 1). A binarized explicit mask of the cerebellum was used for all second-level analyses.

To investigate connectivity patterns between the cerebellum and cerebrum, the gPPI approach was used. For this, realigned and slice-time corrected whole-brain images were additionally normalized to the MNI template (voxel size 3 mm isotropic). The resulting images were smoothed with an isotropic 8 mm FWHM and forwarded to the first-level analyses which followed the procedure and settings described above (i.e., regressors, motion-correction, AR1, high-pass filtering). A 4-mm sphere built around the activation peak found in lobule VIIIa for the interactional contrast SPD – CG: fast – slow served as the seed region.

All analyses (activity/connectivity) used probabilistic region-of-interest (ROI) masks with a 50% threshold. Cerebellar masks were taken from the SUIT atlas. This resulted in the following cerebellar masks (Crus I/II, IV, V, VI, VIII a/b, IX, X) for each hemisphere and an additional mask for the vermis. All other ROI masks were taken from the Harvard-Oxford cortical and subcortical structural atlases. Results were considered significant if p < .05 corrected for family-wise error (FWE) on the voxel level (and cluster size > 10 voxels). All ROI results are small volume corrected.

### Analysis of rating data

A repeated-measures analysis of variance (ANOVA) tested the effects of TOUCH (slow, fast), and GROUP (CG, SPD) on ratings for valence, arousal, and urge to pick one’s skin. Reported effect sizes are eta squared (η²).

## Results

### Demographic data

The two groups did not differ in mean age (t(130) = -1.55, (p = .12), and handedness (χ² = 2.77, p = .25; see Table 1). Since the majority of individuals were right-handed (n = 113 (86%), a comparison between left/right-handed participants was not possible. Most of the participants had at least a high-school diploma (SPD: n = 68 (97%), CG: n = 60 (97%)).

**Table 1:** Comparison of the skin-picking (SPD) group and the control group (CG)

*Demographic data*		SPD (n = 70 females) M (SD)	CG (n = 62 females) M (SD)	
Age (years)		25.57 (6.82)	23.87 (5.59)	
Handedness (n; R/L)		61/7	52/10	
***Rating data***				*Total*
**Valence**				
Slow		6.19 (1.68)	7.22 (1.27)	6.67 (1.58)
Fast		3.85 (1.52)	5.01 (1.81)	4.40 (1.76)
Total		5.02 (1.19)	6.12 (1.36)	
**Arousal**				
Slow		3.15 (1.30)	1.78 (0.75)	2.51 (1.28)
Fast		4.11 (1.56)	2.28 (1.27)	3.25 (1.69)
Total		3.63 (1.19)	2.03 (0.88)	
**Urge to pick**				
Slow		3.14 (1.60)	1.32 (0.65)	2.30 (1.55)
Fast		3.79 (1.80)	1.61 (0.99)	2.74 (1.83)
Total		3.46 (1.42)	1.46 (0.73)	

Footnote: M (mean); SD (standard deviation); slow = brushing velocity 3 cm/s; fast: brushing velocity 30 cm/s;

### Motion characteristics

Total displacement did not differ between the SPD group (M: 1.34, SD: 0.80) and the Control group (M: 1.27, SD: 0.83; t(126) = − 0.47, p = .642). Additionally, there was no statistically significant correlation between total displacement and the rating data in the total sample (all ps > 0.140, range r: -0.04 to 0.13).

### Questionnaires

The SPD group obtained higher scores M (SD) on the SPS-R (SPD: 16.36 (2.88) vs. control: 1.42 (1.95); t = 35.16), MIDAS_focused picking scale (SPD: 23.61 (3.93) vs. control: 8.68 (3.89); t = 25.54), and MIDAS_automatic picking scale (SPD: 18.80 (4.79) vs. control: 14.84 (3.91); t = 21.92) than the control group (df = 82.56-129.16; all p < .001).

### Ratings

*Valence*: The effects of TOUCH (F1,130) = 190.24, p < .001, η² = 0.32) and GROUP were significant (F1,130) = 24.61, p < .001, η² = 0.07). Slow touch was experienced as more pleasant than fast touch (mean difference (Δ): 2.27). The SPD group felt less pleasant than the control group (Δ: -1.1) while being touched. The interaction GROUP x TOUCH was not statistically significant (F(1,130) = 0.15, p = .698, η² = 0.00).

*Arousal*: The effects of TOUCH (F(1,130) = 35.54, p < .001, η² = 0.06) and GROUP were significant (F(1,130) = 76.09, p < .001, η² = 0.27). Fast touch was experienced as more arousing than slow touch (Δ: 0.74). The SPD group reported higher arousal than the control group (Δ: 1.6). The interaction GROUP x TOUCH was not statistically significant (F(1,130) = 3.50, p = .064, η² = 0.01).

*Urge to pick one’s skin*: The effects of TOUCH (F1,130) = 13.28, p = .001, η² = 0.02) and GROUP were significant (F1,130) = 99.9, p < .001, η² = 0.34). Fast touch was associated with a greater urge to pick relative to slow touch (Δ: 0.44). The SPD group reported a greater urge to pick the skin relative to the control group (Δ: 2). The interaction GROUP x TOUCH was not statistically significant (F1,130) = 2.07, p = .15, η² < 0.001). Detailed information about means and standard deviations can be found in Table 1.

### Brain activity

Relative to the control group, the SPD group showed reduced activity in the right lobule VIIIa (MNI coordinates: 30,-58,-49, t-value: 3.70, p(FWE) = 0.033) during slow vs. fast brushing.

*Correlation findings for the contrast slow vs. fast brushing*: In the SPD group, the difference scores for the urge to pick ratings (slow – fast) were positively correlated with activity in the right Crus II (MNI coordinates: 46, -50, -43, t-value: 3.86, p(FWE) = 0.040), right lobule VIIIb (MNI coordinates: 14, -54, -63, t-value = 4.34, p(FWE) = 0.005) and left lobule VIIIa (MNI coordinates: -8, -70, -43, t-value = 4.04, p(FWE) = 0.018). In the control group, the correlation was negative.

**Fig. 1:**
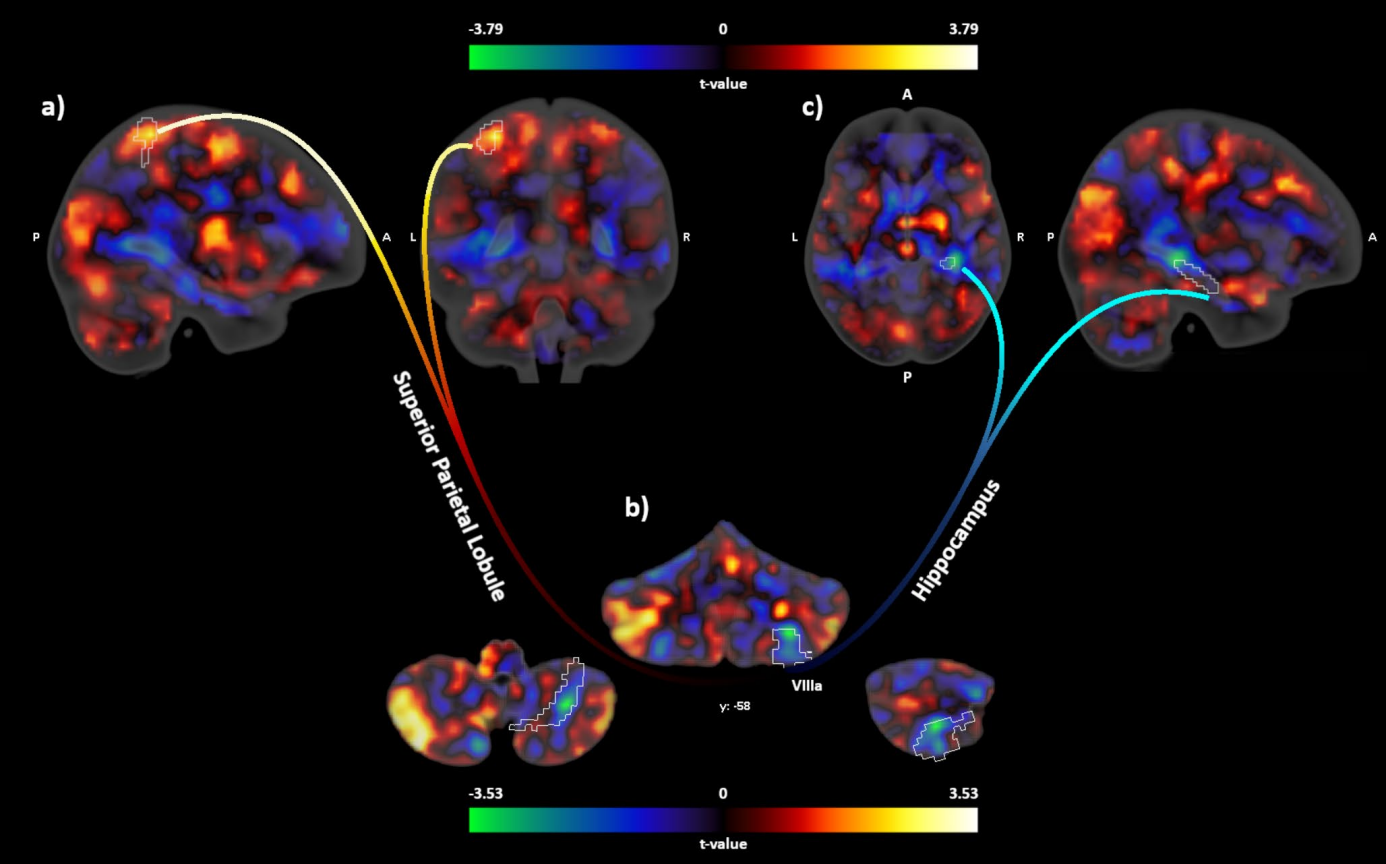
Unthresholded cerebellar activity and connectivity maps (contrast: slow – fast brushing). **a**) Increased connectivity between lobule VIIIa and the superior parietal lobule in patients with SPD relative to controls, **b**) Decreased lobule VIIIa activity in patients with SPD relative to controls, **c**) Reduced connectivity between lobule VIIIa and the hippocampus in patients with SPD relative to controls

Concerning the valence difference scores (slow - fast), the SPD group showed a negative correlation with activity in the left Crus I (MNI coordinates: -40, -60, -25, t-value: 4.08, p(FWE) = 0.029), left lobule VI (MNI coordinates: -38, -58, -25, t-value: 4.19, p(FWE) = 0.014) and right lobule V (MNI coordinates: 8, -62, -17, t-value: 3.60, p(FWE) = 0.042). In the control group, the correlations were positive. Scatterplots of the findings are depicted in Supplementary Figure S1. The correlation analysis for the arousal ratings revealed no group differences. Figure 1

### Functional connectivity: slow vs. fast brushing

Relative to the control group, the SPD group showed reduced connectivity between the right lobule VIIIa (seed) and the right hippocampus (MNI coordinates: 33, -34, -4, t-value: 3.75, p(FWE) = 0.01) and increased connectivity between lobule VIIIa and the left superior parietal lobe (MNI coordinates: -30, -46, 62, t-value: 3.00, p(FWE) = 0.049).

## Discussion

The present analysis adds to the limited knowledge concerning cerebellar involvement in skin-picking disorder (SPD). We focused on touch processing because this sensory modality is directly related to the symptoms of SPD (repeated touching/ picking of the skin that is typically perceived as pleasant).

Firstly, the SPD group showed reduced activity in lobule VIII compared to healthy controls. Lobule VIII is part of the posterior cerebellum that has sensorimotor and cognitive functions (see meta-analysis by Stoodley & Schmahmann, 2009). Lobule VIII activation has been identified previously in a study that implemented a similar design to the present investigation (however not in the context of SPD; Bushara et al., 2001). In that study, soft/slow stroking using a tongue depressor tip was applied to the hands of participants. The stroking activated distinct areas in the anterior and posterior lobes of the cerebellum including lobule VIII. This finding can be interpreted in light of the motor control functions of VIII, which is involved in fine motor coordination and inhibition of (involuntary) movement (Stoodley & Schmahmann, 2010). If someone is gently touched by another person, this does not require a motor action. Affective touch can be passively enjoyed. Therefore, movement should be inhibited. The present data with reduced VIII activity during affective touch in patients with SPD might reflect difficulties concerning such inhibitory processes. This interpretation is consistent with previous neuroimaging studies that revealed abnormal activity in brain regions concerned with motor control in patients with SPD, such as the IFG and ACC (e.g., Grant et al., 2022).

Further, an investigation (without fMRI) by Schienle and Wabnegger (2022) already pointed to dysfunctional motor responsivity related to SPD. In this study, soft brushing of participants’ forearms elicited an increased urge to pick the skin in those with elevated skin-picking severity. Thus, individuals who excessively picked their skin showed a paradoxical reaction to affective touch; after the soft tactile stimulation that is typically perceived as pleasant and calming, they had the urge to scratch their skin. Seen in the context of the findings in the current study, the reduced lobule VIII activity in individuals with SPD might be related to problems in inhibiting motor responses (skin-picking). In line with this assumption, a study by Grodd et al. (2001) demonstrated that lobule VIII is concerned with the execution and control of voluntary movements of the arm (and face). Moreover, the authors of that study demonstrated the involvement of lobule VIII in the somatotopic representation of the arm/hand region. It is also possible that alterations in the sensorimotor topography (e.g., concerning the representation size for different body parts) might contribute to the skin-picking symptoms in patients with SPD. Qualitative studies in which participants are asked to describe the specific sensations elicited by the soft/slow stroking of their arms (e.g., prickling, tingling sensations) might be helpful to better understand cerebellar dysfunctions in SPD that are associated with tactile processing.

In addition to sensorimotor functions, the right posterior cerebellum is involved in cognitive (executive) functions (see Timmann et al., 2010). For instance, research has identified bidirectional connections between the cerebellum and the hippocampus (e.g., Yu et al., 2015). This functional connectivity has important implications for cognitive functions such as tasks requiring the use of self-motion information; for example, in a study by Igloi et al. (2014), cerebellar-hippocampal coactivation was found during egocentric navigation. In addition, timing-dependent motor tasks rely on the information exchange between the cerebellum and hippocampus (Yu et al., 2015). These observations appear to be in line with the connectivity findings of the present experiment, which indicated an altered functional coupling of lobule VIII with the hippocampus in the SPD group. The reduced coupling in SPD patients possibly indicates difficulties in decoding temporal characteristics of touch and suggests that the ability to differentiate between slow vs. fast brushing might be limited in patients with SPD. This hypothesis should be directly tested in a future investigation, where different brushing velocities should be administered.

Another finding in the current study was related to the functional connectivity between lobule VIII and the superior parietal lobule (SPL), which was enhanced in patients with SPD. The SPL plays an important role in cognitive functions (e.g., attention, working memory; Koenigs et al., 2009) as well as visuomotor functions. For example, the SPL is activated during the execution of motor actions, observation of motor actions, and mental simulation of action (Grezes & Decety, 2001). The enhanced lobule VIII-SPL coupling in SPD patients seen in the current study might therefore be associated with the planning/ preparation of performing the motor action of skin-picking. However, this result should be treated with caution as the observed effect was rather small (p = .049).

The self-report data indicated that participants with an SPD diagnosis experienced the tactile stimulation (both fast and slow brushing) as less positive and more arousing than the control group, and experienced a greater desire to pick their skin. Thus, the rating data did not completely mirror the neural data that indicated specific dysfunctions related to affective touch processing in SPD. The affective ratings were associated with the activation of ‘limbic’ cerebellar regions (Crus, lobule VI, VIII; Schraa-Tam et al., 2012). Greater Crus activation was associated with a more negative affective state and a greater urge to pick the skin in patients with SPD. In line with this finding, Crus activity has repeatedly been observed during emotion processing (Stoodley & Schmahmann, 2009; Guell et al., 2018). Moreover, a meta-analysis by Pierce et al. (2022) identified activity in lobule V/VI across different affective tasks (in addition to the involvement of Crus I/II). In the current study, activation within lobule VI was negatively correlated with the valence ratings for affective touch in the patient group. In essence, the self-report data from the SPD group indicated a less pleasurable reaction to touch, and this was associated with the activation of cerebellar regions with affective functions. However, we did not identify group differences in these regions.

In summary, the main finding of the current study refers to reduced lobule VIII activity in patients with SPD. This observation is in line with previous studies using behavioral approaches (e.g., Odlaug et al., 2010; Wabnegger & Schienle, 2022), structural neuroimaging (e.g., Grant et al., 2013; Blum et al., 2018), as well as functional neuroimaging (e.g., Odlaug et al., 2016) that described SPD-related deficits in motor control. A future investigation could evaluate Inhibitory Control Training (ICT) for patients with SPD. This training would require patients to respond to environmental stimuli as quickly and accurately as possible unless a different stimulus (Stop Signal/ No-Go cue) is presented (Spierer et al., 2013).

### Electronic supplementary material

Below is the link to the electronic supplementary material.

Supplementary Material 1

## Data Availability

On request from the authors.
