# Supplementary figures and images for: Atypical cerebellar activity and connectivity during affective touch in adults with skin-picking disorder
Source: Brain Imaging Behav. 2023 Nov 17;18(1):184–91. doi: 10.1007/s11682-023-00824-z (PMC10844139; doi:10.1007/s11682-023-00824-z)

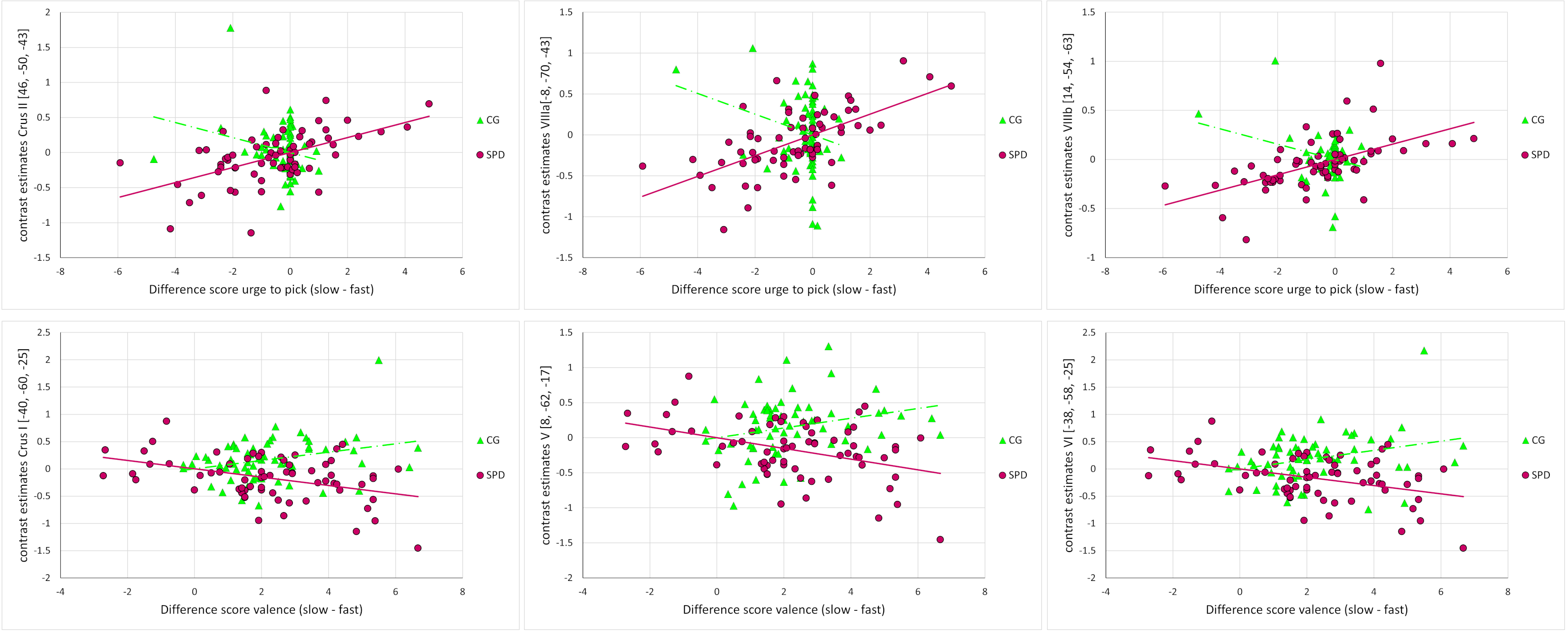

Supplement: Supplementary file 1 — Supplementary Material 1 [file 11682_2023_824_MOESM1_ESM.png]
